# Close of the Volume

**Published:** 1858-12

**Authors:** 


### EDITORIAL.

### CLOSE OF THE VOLUME.

The present number closes the first volume of the *Chicago Medical Journal*, and in it will be found a title page and full index. The only thing we have to regret, in looking over our work, is the fact that during the last four months we have been compelled to issue the Journal at the end instead of the beginning of each month. We shall soon be able to remedy this, however, and we have made such arrangements, both in regard to contributions and illustrations, as will secure to the forthcoming volume a higher degree of interest and value than any which has preceded it. This, with the increased size of each number, we hope will be sufficient to induce all our friends to promptly renew their subscriptions and to send us in addition many new names as subscribers.

